# Therapeutic Potentials of Low-Dose Tacrolimus for Aberrant Endometrial Features in Polycystic Ovary Syndrome

**DOI:** 10.3390/ijms22062872

**Published:** 2021-03-12

**Authors:** Ahmad J. H. Albaghdadi, Frederick W. K. Kan

**Affiliations:** Department of Biomedical and Molecular Sciences, Faculty of Health Sciences, Queen’s University, Kingston, ON K7L 3N6, Canada; a.albaghdadi@queensu.ca

**Keywords:** polycystic ovary syndrome (PCOS), endometrial progesterone resistance, tacrolimus, immunosuppression, chronic inflammation

## Abstract

Polycystic ovary syndrome (PCOS) is a major anovulatory infertility affecting a great proportion of women of childbearing age and is associated with obesity, insulin resistance and chronic inflammation. Poor endometrial receptivity and recurrent implantation failure are major hurdles to the establishment of pregnancy in women with PCOS. The accumulating body of evidence obtained from experimental and clinical studies suggests a link between inherent adaptive and innate immune irregularities and aberrant endometrial features in PCOS. The use of conventional therapeutic interventions such as lifestyle modification, metformin and ovarian stimulation has achieved limited clinical success in restoring ovulation and endometrial receptivity in women with PCOS. Unlike other immunosuppressive drugs prescribed in the clinical management of autoimmune and inflammatory disorders that may have deleterious effects on fertility and fetal development, preclinical studies in mice and in women without PCOS but with repeated implantation failure revealed potential therapeutic benefits for the use of low-dose tacrolimus in treating female infertility. Improved systemic and ovarian immune functions, endometrial progesterone receptor and coreceptor expressions and uterine vascular adaptation to pregnancy were among features of enhanced progesterone-receptor sensitivity in the low-dose tacrolimus-treated mouse model of the disease. In this review, we have compiled available experimental and clinical data in literature on endometrial progesterone resistance and current therapeutic options, as well as mechanisms of actions and reported outcomes relevant to the potential therapeutic benefits for the use of low-dose tacrolimus in treating PCOS-associated female infertility.

## 1. Introduction

Polycystic ovary syndrome (PCOS) is a heterogeneous endocrine disorder and a major cause of anovulatory infertility affecting 6–10% of women of reproductive age [1,2,3]. Human and experimental studies confirm that, in its typical form, this multisystemic hormonal disorder is characterized by oligo- and/or anovulation, as well as hyperandrogenism, and is often associated with obesity, insulin resistance, dyslipidemia, immune disturbances and increased risk of cardiovascular events [4]. After excluding other ovarian and endocrine disorders simulating PCOS, the diagnosis of the disease is exclusively dependent on two of three features: irregular menstrual cycles with oligo- or anovulation, and polycystic ovarian morphology (PCOM) together with clinical and/or biochemical evidence of hyperandrogenemia [5,6]. Recommendations from the international evidence-based guideline for the assessment and management of PCOS (i.e., the Rotterdam diagnostic criteria) characterized four phenotypes of the syndrome: (1) Type A (classic) presents with irregular cycles with hyperandrogenism (HA) and polycystic ovarian morphology (PCOM), (2) Type B (classic) presents with HA and irregular cycles, (3) Type C (ovulatory, nonclassical) presents with HA and PCOM, and finally (4) Type D (normo-androgenic) presents with irregular cycles and PCOM [7] (Table 1). Beside hormonal disturbances, particularly hyperandrogenemia and associated chronic anovulation, low progesterone (P4) levels and ensuing oligo-menorrhea further leading to endometrial dysfunctions, loss of endometrial plasticity is believed to be among contributing factors in the development of the reported cyclical irregularities in PCOS [8]. It is generally held that combinations of primary (genetic and cellular) endometrial abnormalities manifesting during prenatal development, together with postnatal endometrial defects secondary to hormonal, metabolic, inflammatory and immune malfunctions, result in these PCOS-associated endometrial dysfunctional phenotypes [9,10]. Indeed, evidence of primary endometrial defects in women with PCOS is demonstrated in the existence of inherent endometrial aberrancies in both ovulatory and anovulatory phenotypes affecting several biological pathways and the anomalous expressions of proteins involved in endometrial receptivity, such as those related to cell adhesion and the cytoskeleton network, transcriptional regulation, DNA repair, apoptosis and cell cycle regulation, cellular transport and signaling and mitochondrial metabolism [11,12,13,14,15]. Importantly, evidence of perturbed endometrial immune responses and poor endometrial receptivity has also been reported in ovulatory and anovulatory women with PCOS [10,11,16,17].

## 2. Evidence of Endometrial Immune Irregularities in PCOS

Despite a plethora of emerging data and myriad suggested hypotheses on the immunological abnormalities associated with PCOS, the exact mechanism(s) underlying the immune etiology of this endocrine metabolic disorder and its links to disturbed endometrial functions remain moot. To date, the general consensus is that the onset of PCOS is marked by low-grade inflammation characterized by elevated serum levels of CRP, IL1, IL6 and products of activated monocytes (e.g., TNFα, IFNγ, MCP1, MCP5 and MIP) as well as molecules originated from leukocyte–endothelium interactions (e.g., VCAM-1, ICAM-1) [19,20,21]. Autoantibodies have also been detected in women with PCOS [22,23], and a causal association between chronic low-grade inflammation and the age- and adiposity-dependent development of endocrine, metabolic and cardiovascular adversities in PCOS has been reported [24,25]. At the endometrial level, impaired progesterone-mediated decidualization of endometrial stromal fibroblasts was associated with aberrant pro-inflammatory gene-expression profiles in the form of increased expression of IL8, monocyte chemo-attractant proteins MCP1 and MCP3 (CCL2 and CCL7, respectively), RANTES (CCL5) and granulocyte-macrophage colony-stimulating factor (GM-CSF) [17]. These have been reported among several endometrial cell populations in the proliferative phase endometrium of anovulatory women with PCOS [10,17]. Moreover, proteomic studies by Rashid et al. (2020) unraveled evidence of aberrant differential expression of proteins involved in inflammation, humoral immune responses, immunomodulation and immune-checkpoint mechanisms in women with PCOS with subdued fertility and dampened endometrial receptivity [11]. These findings are supported by the significant relationship between programmed cell death-1 (PD-1) and programmed cell death-1-ligand I (PD-L1) polymorphisms and PCOS, which was reported by Han et al. (2021) [16]. Altogether, the importance of the findings by Rashid et al. (2020) and Han et al. (2021) stems also from the critical role of the PD-1/PD-L1 immune-checkpoint pathway in modulating the cytotoxic activity of a subset of decidual CD8+ T cells, namely the PD1/NKG2D double positive CD8+ regulatory T cells (Tregs), which contribute to maternal immunotolerance at the maternofetal interface [26]. Another important subset of endometrial Tregs involved in modulating local immune milieu during implantation are the CD4+CD25+ T cells [27]. The quintessential role of these fetal-specific Tregs in suppressing the allogeneic response against the implanting embryo and supporting early gestation is well demonstrated in previous animal studies [28,29,30]. Whereas adaptive transfer of exogenous Tregs in the peri-implantation period in depleted mice prevented fetal loss [29], targeted depletion of these T cells during implantation terminates pregnancy [28]. One major mechanism for the differentiation of these Tregs from their naïve CD4+ status is the activation and expression of the fate-determining transcriptional factor FoxP3, which are believed to be achieved under the influence of the modulatory cytokines TGF-β, IL2 and IL15 [27,31]. This critical differentiation of the CD4+ T cells into functioning FoxP3+ CD4+CD25+ Tregs might be defective in PCOS [32]. A study by Krishna et al. (2015) reported that the inability of the CD4+CD25+ T cells, extracted from the entire peripheral blood mononuclear cell population, to expand during the follicular phase is most likely a consequence of an inherent hyporesponsiveness to IL2 signaling and compromised STAT5 activation in women with PCOS [32]. STAT5 is necessary for Treg development, as it binds to the FoxP3 promoter and regulates FoxP3 expression [33]. Preclinical studies in mice have also revealed that the declining thymic ability of the estrogen-primed female mouse to produce CD4+CD25+ regulatory T cells culminated in anovulation and ovarian-cyst development [34]. Moreover, among features of perturbed Tregs functions in PCOS is the low-level expression of the leukemia inhibitory factor (LIF) in the endometria of women with PCOS [35]. LIF is a Treg cytokine that influences fate determination in these cells [36,37]. More importantly, animal studies and clinical data support a critical role for LIF in preparing the endometrium for embryo implantation and postimplantation embryonic development [38,39]. Therefore, the crucial role of FoxP3+ Tregs in endometrial receptivity and embryo implantation [30,40,41] dictates the need for alternative therapeutic approaches to support endometrial FoxP3 activation and the periconceptional expansion of the CD4+CD25+ Tregs in infertile women [27], especially those with PCOS.

## 3. Evidence of Endometrial Progesterone Resistance in PCOS

A resistance to the hormonal actions of progesterone (P4), also referred to as P4 resistivity, has been described in women with infertility, including those with PCOS [42,43,44]. Human studies established that PCOS endometrium is inherently different with respect to its response to progesterone [44,45]. PCOS patients exhibit poor endometrial receptivity and reduced effectiveness and exposure to progesterone [46]. Molecular mechanisms associated with the latter are not well understood. However, quantitative and qualitative alterations in the endometrial epithelial progesterone receptor (PGR) have been described in the secretory endometrium of women with PCOS [46,47,48]. Quezada et al. (2006) confirmed that higher levels of expression of the endometrial epithelial PGR messenger RNA (mRNA) and protein are associated with an aberrant higher expression of the androgen receptor associated protein 70 (ARA70) and low level of the adhesion molecule beta-3 integrin in the midsecretory endometrium of women with PCOS [46]. Aberrancies in the endometrial epithelial expressions of PGR-A and PGR-B have also detected in the proliferative phase of chronically anovulatory obese women with PCOS [47]. Studies by Paulson et al. (2017) revealed that the aberrant overexpression of the endometrial epithelial and stromal PGR-B and the downregulation of the PGR-A in the proliferative endometrium of obese women with PCOS likely impacted proliferation of the secretory-phase endometrium [47]. Features of defective endometrial progesterone actions have also been confirmed by Hu et al. (2018) [48], suggesting that the aberrant overexpression of PGR-B in the cytoplasm rather than the nuclei of the endometrial epithelial and stromal cells is associated with the development of PCOS in women [48]. The culprit of the abovementioned irregularities of the endometrial PGR expression and localization is believed to be, in part, due to chronic anovulation and the elevated levels of androgens and their receptors, estrogen receptor alpha (ERα) and steroid receptor co-activators in the PCOS endometrium [48,49]. However, the plausible mechanisms involved in this progesterone resistance may reside in the progesterone receptor (PGR) itself. This was first reported by Chrousos et al. (1986) [50] and is supported by the work of Igarashi et al. (2005) [51] establishing that alteration in the pattern of endometrial PGR expression leads to endometrial P4 resistivity [51]. According to their cellular locations and mechanisms of actions, progesterone receptors are either located at the cell membrane, also referred to as PGRM with its two identified component isoforms: PGRMC1 and PGRMC2 conveying the rapid non-genomic actions of P4, and the cytosolic/nuclear PGR (PGR-A and PGR-B) mediating the genomic actions of the hormone [52]. Aberrancies in the expressions of PGR (A and B), PGRMC1 and PGRMC2 in the endometria of anovulatory women with PCOS have been reported [47]. Compared to the control group of regularly cycling women, Paulson et al. (2017) [47] observed persistent higher expressions of PGR (A and B), PGRMC1 and PGRMC2 in the stroma and of PGRMC1 in the luminal epithelium on cycle days 21–23 in obese anovulatory women with PCOS [47]. These findings suggest inherent defects in the cytosolic and nuclear PGR functions and expression in women with PCOS. The cytosolic/nuclear PGR is a heterodimer of the naïve progesterone receptor protein in dynamic interactions with its chaperones HSP90, HSP70 and p23, and its cochaperone immunophilins FKBP51 and FKBP52 [53,54,55,56]. Ligand binding causes the release of multiple HSP-subunit complexes, allowing the PGR to undergo certain conformational changes assisted by the enzymatic action of the FKBPs, which permits the receptor dimer to interact with specific progesterone response elements localized at the regulatory regions of its target genes [57,58]. Studies showed that alterations in the expression or function of PGR coactivators, chaperones, and cochaperones that are bound to the mature form of the cytosolic PGR before activation and nuclear translocation have been implicated in the development of P4 resistivity [59,60,61]. Although few investigations have been conducted on the impact of FKBPs on PCOS, the general consensus is that lack of cochaperone FKBP52 or the overexpression of the cochaperone FKBP51 causes PGR signaling irregularities and endometrial progesterone resistance in experimental murine models [61,62]. Furthermore, among the critical events involved in the generation of a receptive endometrium are the dynamic interactions between endometrial PGR and other transcriptional factors such as the Forkhead box protein O1 (FoxO1) [63,64]. Human and mouse studies confirmed the existence of a vital molecular switch mechanism represented by the reciprocal relationship between endometrial epithelial PGR and FoxO1 expression and activation during the window of receptivity [63,64]. FoxO1 protein is a key mediator of insulin action on gene expressions [65] and is a negative regulator of cell survival that inhibits cell proliferation and promotes cell apoptosis and cycle arrest [66]. Aberrant overexpression and activation of FoxO1 have been reported in women with PCOS [67,68]. Studies by the Vega group suggested a mechanistic link between a persistent high level expression of the phosphorylated FoxO1 (p-FoxO1Ser319) in the endometrial epithelial compartment and disturbed endometrial homeostasis, steroid bioavailability and failed uterine receptivity in obese and hyperinsulinemic women with PCOS [46,68]. Additionally, the aberrantly upregulated FoxO1 was also found to mediate the production of proinflammatory cytokines that alter the PGR expression, such as IL1β, IL6 and TNF-α, in women with PCOS [67]. While these reports may suggest a role of abnormal FoxO1 signaling in the development of restricted progesterone-dependent uterine receptivity and decidualization in PCOS, further molecular studies into the mechanistic association between the endometrial PGR and other transcriptional mediators of endometrial receptivity, particularly the Indian hedgehog (IHH) signaling pathway, are warranted. Notably, the fact that the persistence of the abovementioned endometrial irregularities despite adequate metabolic control, lifestyle interventions and the restoration of regular ovulatory cycles [47,69] underpins the need to further examine the link between chronic inflammation and related immunological abnormalities in the development of P4 resistivity in PCOS.

## 4. Current Therapeutic Options for PCOS

Current consensus guidelines and clinical accord on the management of anovulatory infertility in women with PCOS focus largely on improving ovulatory function and managing oligo- or anovulation-related subfertility [5,70]. Therefore, irrespective of their FDA-approval status, current therapeutic interventions for PCOS include lifestyle modification and weight management, as well as drugs that induce ovulation, such as clomiphene citrate (CC) and aromatase inhibitors (such as letrozole and/or anastrozole) [71,72,73,74]. Additionally, combined oral contraceptive pills (OCPs) have been used to improve hyperandrogenism in women with PCOS [75], and for its demonstrated clinical and metabolic benefits improving ovulation and menstrual frequency in anovulatory PCOS patients, the insulin-sensitizing agent metformin has also been prescribed [76]. Weight loss is considered an initial treatment strategy for reproductive disorders in overweight and obese women with PCOS [25]. Evidence showed that active lifestyle management, weight loss and physical activity help menstrual disturbances and can shorten the time to conception and reduce adverse obstetric risks [77]. However, the current management of the irregular menstrual cycles to protect the endometrium against development of hyperplasia entails the use of OCPs [78]. The flip side of the use of OCPs in PCOS not only resides in the increased risk of inflammatory and coagulatory disorders and cardiovascular-disease development [79,80], but also in the lack of sufficient data from randomized clinical trials comparing progestins alone versus combined estrogens and progestins in the treatment of irregular vaginal bleeding due to anovulation. This has cautioned the need for alternative therapeutic approaches [81].

Among insulin-sensitizing agents primarily used to treat insulin resistance with demonstrated clinical and metabolic benefits in the management of PCOS in women is metformin [82,83]. Although metformin is not approved by the US Food and Drug Administration (FDA) for the treatment of infertility in women with PCOS [84], its use in PCOS is largely derived from case-control studies with occasional conflicting results [85,86,87]. A prospective evaluation of the safety of metformin administration during pregnancy in 98 women with PCOS done by De-Leo and colleagues (2011) [86] found a significant reduction in pregnancy complications such as gestational diabetes mellitus (GDM) and gestational hypertension [86]. On the other hand, data obtained from a randomized double-blind placebo-controlled trial conducted by Vanky and associates (2010) [85] revealed no difference in the primary outcome, which was a composite of preeclampsia, spontaneous abortion, GDM and preterm delivery among 257 women with PCOS receiving metformin (500–1000 mg twice daily) from the first trimester to delivery [85]. Nonetheless, besides its systemic and ovarian actions in reducing peripheral insulin resistance, regulating hepatic glucose release and inhibiting androgen production in the ovaries, experimental and clinical data showed that metformin may have a direct modulatory effect on the endometrium [83,88,89]. Experimental studies revealed that metformin could ameliorate uterine receptivity defects and partially improve implantation rates, and had significant immunosuppressive properties when used at a high dosage in the treatment of PCOS-associated adverse pregnancy outcomes in murine models of the disease [90,91]. In doing so, the molecular mode of action of metformin on the endometrium seems to be diverse. A study by Zhai et al. (2019) [92] suggested that metformin may improve endometrial receptivity through increasing the expression of the homeobox A10 (HOXA10) and integrin beta-3 (ITGB3) via the downregulation of miR-1910-3p and miR-491-3p in the endometrium of women with PCOS [92]. These findings are supported by recent data from Zhang et al. (2020) [93] on the modulatory effect of metformin on the activation of the AMPK signaling pathway in the endometrium of diabetic pregnant mice in improving implantation rates [93]. Moreover, in a study reported by Xiong et al. (2019) [94], metformin was found to alleviate estradiol and progesterone-induced decidualization of human endometrial stromal cells by modulating the secretion of multiple cytokines, inhibiting expression of matrix metalloproteinase-2 and -9, activating MAPK/ERK/p38 signaling and reducing PGR expression [94]. Metformin can also normalize androgen-receptor-mediated transcriptions, thereby restoring endometrial epithelial–stromal crosstalk, and has significant antagonizing actions in reversing the androgen-induced alterations in the insulin receptor substrate and GLUT4 expressions in endometrial glandular epithelial cells [95,96]. However, notwithstanding the additional health benefits for the use of metformin in reducing serum atherogenic biomarkers, such as the advanced glycation end products and C-reactive protein [79], the role of metformin in treating PCOS is narrowing [97]. Metformin may improve the menstrual cycle within 1–3 months; nonetheless, multiple reports from systemic reviews and randomized clinical trials indicate that metformin may not improve implantation and/or live birth rates or reduce miscarriage in women with PCOS [98,99,100]. More importantly, the use of metformin during pregnancy did not reduce maternal weight gain or avert gestational diabetes mellitus when initiated during pregnancy in at-risk women [101,102], nor did it reduce the relatively high obstetrical risk associated with increased rates of cesarean sections and the delivery of babies that were large for their gestational age and macrosomic babies (i.e., >4 or 4.5 kg at birth) [101,102,103].

Unlike metformin, the use of ovulation-induction agents such as the aromatase inhibitor letrozole has gained popularity [104,105,106]. Letrozole is effective for ovulation induction, and new data show improved live birth rates by skipping a progestin withdrawal bleed and proceeding directly with a dose escalation of letrozole in clomiphene citrate (CC)-resistant women with PCOS [77,106]. However, unlike CC, letrozole is categorized as pregnancy class D drug and is not FDA-approved for treating infertility and inducing ovulation [74]. Nonetheless, experimental and clinical data have indicated better effects of letrozole compared to CC on endometrial thickness and the expression of endometrial receptivity markers, and those associated with embryo implantation and placental development such as the Wnt-beta-catenin pathway critical for embryo implantation [107,108]. All in all, the safety and efficacy of letrozole or clomiphene citrate in achieving live birth in infertile women with PCOS will need to be further evaluated.

Other therapeutic modality in the management of PCOS-associated female infertility is the use of gonadotropins [77,109]. Ovulation induction with follicle-stimulating hormone (FSH) is currently reserved as a second-line treatment for anovulatory women with PCOS who fail to respond to CC or letrozole [77]. Irrespective of the source and clinical formulation, clinical data and meta-analyses indicated little or no difference in live birth rates, multiple pregnancy rates, clinical pregnancy rates or miscarriage rates between urinary-derived gonadotropins and recombinant FSH in women with PCOS [109]. Moreover, data obtained from large multicenter randomized clinical trials, such as the Assessment of Multiple Intrauterine Gestations from Ovarian Stimulation (AMIGOS) trial involving a cohort of 900 couples with unexplained infertility that included women with PCOS, supported a higher efficacy of gonadotropins compared to CC or letrozole for ovulation induction [110]. More important is the downside for the use of gonadotropins, which is higher rates of multiple pregnancies compared to CC or letrozole, which is hard to predict using clinical or biochemical markers of androgenic activity such as the serum concentrations of androgens [110,111]. A list of current pharmacological agents commonly used for treating anovulatory infertility in women with PCOS is summarized in Table 2.

## 5. Mode of Action of Tacrolimus and Its Potentials to Mitigate Progesterone Resistance and Associated Menstrual and Endometrial Abnormalities in PCOS

Tacrolimus (FK506) is a lipophilic immunosuppressant with a 23-membered ring macrolide macrolactam structure [127] that is firmly established in the clinical routine of immunosuppression. Recently, tacrolimus has been successfully prescribed in the preclinical management of PCOS-related female infertility in an obese murine model of PCOS [128] and in treating women without PCOS but with recurrent implantation failure (RIF)/recurrent pregnancy loss (RPL) with elevated systemic Th1 (CD4^+^ IFNγ^+^):Th2 (CD4^+^IL4^+^) cell ratios [129]. The first identified mode of action of tacrolimus is the inhibition of the T-cell receptor (TCR)-mediated Ca^++^-dependent activation of the nuclear transcriptional factors NFκB and the nuclear factor of activated T cells (NFAT) [130]. This action is primarily contingent upon the binding of tacrolimus (FK506) to the FK506-binding protein 12 (FKBP12) and the subsequent inhibition of the phosphatase activity of the Ca^++^-dependent serine/threonine (Ser/Thr) protein phosphatase calcineurin, thereby suppressing the dephosphorylation and nuclear translocation of NFκB and NFAT in a dose-dependent manner [131,132,133,134,135]. The primary outcome of this tacrolimus-suppressed TCR signaling is the restricted release of INFγ and IL2, as well as TNFα and GM-CSF, in activated peripheral blood monocytes and plasmacytoid dendritic cells in a dose-dependent manner [136,137]. Importantly, binding of tacrolimus to other members of the tetratricopeptide repeat (TPR) domain-containing FKBPs, particularly FKBP52, has been identified and has proven to influence varieties of physiological functions, particularly those mediated by the glucocorticoid receptors (GR) [138,139]. It has been shown that this binding of tacrolimus to the GR/cochaperone complex could increase the GR receptor transactivity and hormone-binding affinity [139,140]. These TPR cochaperones exhibit peptidylprolyl cis-trans isomerase (PPlase) activity, which, upon activation through the binding of tacrolimus, allows for the release of the mature form of the glucocorticoid receptors from the complex and their subsequent nuclear translocation for the mediation of their genomic actions [141,142]. In fact, the critical role of FKBP52 in the nuclear translocation of steroid receptors has been documented in a variety of cellular contexts [58,143]. The cytoplasmic fraction of FKBP52 is localized to microtubules and serves as an adaptor between dynein, which is attached to the PPlase domain of the protein and the TPR domain-bound GR/HSP90 complex of the steroid receptor [58,144]. Davis et al. (2002) [145] reported a switch mechanism in which the hormone causes exchange of the inhibitory cochaperone FKBP51 for the stimulatory FKBP52 in the GR complex [145]. This exchange has also been shown to influence the corecruitment of the dynein motor protein and movement of the mature GR complex to the nucleus for downstream genomic signaling [145].

Moreover, tacrolimus can modulate the transcriptional activities of the PGR through mechanisms involving the activation of the protein inhibitor of activated STAT-y (PIASy) [146]. PIASy is a member of the PIAS family E3-type small ubiquitin-like modifiers (SUMO) capable of transcriptionally repressing the PGR receptors directly through SUMOylation or indirectly via binding to the steroid receptor DNA-binding domain [147]. We have previously shown that PIASy is aberrantly downregulated and that it has low affinity to be recruited to the nuclear PGR in the peri-implantation uterus of an obese mouse model of PCOS [146]. We have also demonstrated that the use of low-dose tacrolimus rescued the endometrial expression of PIASy and restored its binding capacity to interact with and regulate the transcriptional activity of the nuclear PGR in these uteri [146]. Previous studies showed that the SUMOylation of the PGR, particularly PGR-B, by members of the E3-ligaeses destabilizes its nuclear retention, thereby repressing its transactivation and downstream genomic signaling [148]. Additionally, this PIASy-mediated alterations of PGR phosphorylation rate and the subsequent reduction in its nuclear shuffling and retention efficiency are also mediated by the concerted actions of the histone deacetylases, which bind to active DNA-binding sites, such as those abundantly located within the hinge region of the PGR-A [149,150]. This PIASy-controlled downregulation of PGR-A is histone deacetylases-1-mediated in ex vivo cultured human primary myometrial cells [151]. This is important to our understanding of the mode of action of tacrolimus in restoring endometrial receptivity in PCOS. Both PGR-A and PGR-B were aberrantly expressed during the window of receptivity in the uteri of obese mice with PCOS [146]. The prepregnancy administration of low-dose tacrolimus restored normalized expressions of PGR-A and PGR-B and their interactions with PIASy at the implantation window conducive to gestational success in these mice [146]. This was associated with increased implantation and live birth rates among treated mice [90,146]. Therefore, we believe that through these PGR-regulatory effects, the use of low-dose tacrolimus suppresses heightened endometrial progesterone resistance, promotes endometrial receptivity and aids in the prevention of implantation failure in a murine model of PCOS [146].

## 6. Tacrolimus and Its Potentials to Prevent Dysregulated Treg Response in PCOS

The interplay between the ovarian follicular cells and the effector arm of the follicular immune system, of which the regulatory T cells (Tregs) are critical effectors [152], is indispensable for the control of ovulation [153]. Residents as well as infiltrating lymphocytes contribute substantially to the cyclic-tissue remodeling of the ovary due to their ability to secrete various inflammatory and immunomodulating molecules [152]. As described earlier, among the immunosuppressive regulatory T cells are those defined by their signature expression of the cell-surface molecules CD4, CD25 and the transcriptional factor FoxP3 [154,155,156]. Notwithstanding the limited sample size reported in the human studies, an accumulating body of evidence from animal and human data implicates evident immunological deficits among circulating and ovarian resident Tregs in the pathogenesis of PCOS [32,128,157]. Of the latter are the reduced expression of FoxP3 and decreased expansion of CD4+CD25+CD127low Tregs due to inherent aberrancies in Interleukin 2 (IL2) signaling in women with PCOS [32]. This is valuable to our understanding of the usefulness of low-dose tacrolimus (i.e., ≤10 ng/mL) in mitigating these inherit immunological deficits in PCOS. Firstly, tacrolimus has the potential to bidirectionally regulate the transcriptional activities of FoxP3 in a variety of cellular contexts [158]. Shen et al. (2011) [158] confirmed that this tacrolimus-mediated action on FoxP3 is dose-dependent, and a low concentration of 10 ng/mL tacrolimus resulted in higher nuclear shuffling of the nuclear factor of activated T cells (NFAT) [158]. Members of the NFAT family expressed by the immune cells, including NFAT1, NFAT2 and NFAT4, directly bind to the FoxP3 enhancer and/or cooperate with Smad3 to activate FoxP3 transcription, thereby regulating the production of cytokines by T cells [159]. These diversified actions of NFAT on FoxP3 transcription are contingent upon their calcineurin-mediated dephosphorylation, subsequent nuclear translocation and unmasking of at least six of their T-cell-specific FoxP3 nuclear localization sequences, which positively regulate the transactivation of FoxP3 gene after triggering of the T-cell receptor (TCR) [160,161]. While translating the actions of low-dose tacrolimus on FoxP3 activation in PCOS awaits further investigations, we believe that, at least in part, through this plausible stimulatory effect on FoxP3, low-dose tacrolimus is capable of inducing the periconceptional expansion of the CD4+CD25+CD127low Tregs in a murine model of PCOS [128]. Second, tacrolimus has the potential to moderate the severity of the compromised cross-talks between resident white blood cells, such as macrophages with systemic T helper cells Th1 (CD4+ IFNγ+), Th2 (CD4+IL4+) and Th17 (CD4+IL17A+) [162,163] critically involved in the process of expulsing the oocyte from the antral/Graafian follicles and maintaining a fetal-protective decidual immune milieu during gestation [30,152]. These modulatory effects of low-dose tacrolimus on the expression and activation profiles of the Th1 (CD4+ IFNγ+)/Th2 (CD4+IL4+) and Th17 (CD4+IL17A+) Tregs have indeed been proven effective in the clinical management of women without PCOS but with recurrent implantation failure [129] and in a murine model of PCOS [128], respectively.

## 7. Conclusions

Provision of the best care for women with PCOS requires thorough understanding of the underlying immune and molecular mechanisms associated with poor ovarian functions, heightened progesterone resistance and declining endometrial receptivity. The persistence of clinical, immunological and histopathological features of endometrial malfunction, despite the use of the most effective ovarian stimulation regimen, dictate the need for further investigations into the fundamental molecular and immunological mechanisms of ailing endometrial health in women with PCOS. Evidence presented in this review suggests that immunomodulation with low-dose tacrolimus may mitigate the severity of PCOS-associated female infertility. The efficacy of tacrolimus to promote endometrial receptivity may reside in its intrinsic ability to regulate the endometrial progesterone receptor signaling while suppressing systemic immune aberrancies and associated endometrial immune irregularities in PCOS. Lastly, while the present data from experimental and human studies point to the relative perinatal safety for the use of low-dose tacrolimus in treating female infertility [90,164], further studies are needed to establish the best-fit tacrolimus-based monotherapeutic interventions in the management of PCOS-associated female infertility.

## Figures and Tables

**Table 1:** Classification of polycystic ovary phenotypes (modified from references [7,18]).

Parameter	Phenotype A	Phenotype B	Phenotype C	Phenotype D
HA	+	+	+	−
OD	+	+	−	+
PCOM	+	−	+	+

Abbreviations: HA: hyperandrogenism, OD: ovulatory dysfunction, PCOM: polycystic ovarian morphology.

**Table 2:** Mode of action and fertility-related side effects of current pharmacological agents * commonly used in anovulatory women with PCOS.

Medication	Mode of Action	Fertility-Related Side Effects
**Clomiphene Citrate**	- Induces ovarian follicular development [112]	- Due to its antiestrogenic effect, endometrial proliferation may be hampered [112,113] - May change cervical mucus characteristics with a consequent reduction in sperm penetration [112,113] - May impair endometrial receptivity [114] - May worsen subendometrial/endometrial vascularization as detected by power Doppler [114] - Its use is associated with an increased cardiac anomaly rate [115,116] - Resistance to CC is fairly common in women with PCOS (~ 15% of women with PCOS may not respond to the maximum dose of CC and are considered resistant to this medication) [78]
**Gonadotropins (recombinant follicle-stimulating hormone (rFSH) or human menopausal gonadotropin (HMG))**	- Stimulates endogenous peak of luteinizing hormone for oocyte maturation and ovulation triggering [117]	- Rate of clinical complications such as ovarian hyperstimulation ranges between 6.67 and 17.78% [118]; nonetheless, hCG therapy for PCOS-associated infertility is considered of high clinical curative value [118]
**Letrozole**	- Induces ovarian follicular growth and development and supports postovulatory corpus luteum without having a premature luteinizing effect on the developing follicles [119] - Supports endometrial proliferation through the stimulation of the Wnt/β-catenin pathway [107] - May improve endometrial receptivity and spiral artery resistivity indices [72,107,120] - Inhibits androgen-induced stimulation of E2 and aromatase p450 in endometrial cells [121]	- May result in elevated serum level of FSH [119] - May induce ovarian cyst formation [119] (not reported in CC-resistant women with PCOS [122]) - Can induce PCOS-like ovarian phenotype in experimental animals [123]
**Metformin**	- Improves glucose intolerance [87,88] - Modulates uterine/endometrial ER and PGR expressional irregularities [89] - Suppresses AR expression and normalizes the AR-mediated gene transcription [91,124] - May improve endometrial receptivity defects and improves uterine vascularity and subendometrial blood flow [125]	- Does not prevent the development of GDM [102] - May not improve live birth rate [126] - May not reduce the risk of PCOS-associated adverse pregnancy outcomes such as spontaneous abortion, gestational hypertension, preeclampsia and placental abruption [85] - Causes increased birth weight [103]

Abbreviations: CC: clomiphene citrate, FSH: follicle-stimulating hormone, GDM: gestational diabetes mellitus, ER: estrogen receptor, PGR: progesterone receptor, E2: estradiol, hCG: human chorionic gonadotropin. * Irrespective of current FDA-approval status.

## Data Availability

This review article did not report any previously unpublished research data. All information contained in this review article can be retrieved online at https://www.ncbi.nlm.nih.gov/ (accessed on 2 February 2021).
